# Risk of Rhabdomyolysis and Kidney Injury after Intensive Exercise. Potential of Novel Biomarkers of Kidney Injury: A Narrative Review

**DOI:** 10.5114/jhk/218100

**Published:** 2026-04-02

**Authors:** Eugenia Murawska-Ciałowicz, Gilmara Gomes de Assis, Maria Ciałowicz, Ewa Bakońska-Pacoń

**Affiliations:** 1 Department of Biochemistry, Gdansk University of Physical Education and Sport, Gdansk, Poland.; 2 Escola Superior Desporto e Laser, Instituto Politécnico de Viana do Castelo, Viana do Castelo, Portugal.; 3 Research Center in Sports Performance, Recreation, Innovation and Technology (SPRINT), Melgaço, Portugal.; 4 Faculty of Physiotherapy, Wroclaw University of Health and Sport Sciences, Wroclaw, Poland.; 5 Physiology and Biomechanics Department, Wroclaw University of Health and Sport Sciences, Wroclaw, Poland.

**Keywords:** rhabdomyolysis, high intensity interval training, CrossFit, acute kidney injury, kidney biomarkers

## Abstract

High-intensity interval training (HIIT), CrossFit®, strength training, and others, develop athletes’ strength, speed, and endurance within a very short period of time, enabling competition at the highest sporting level. At present, they constitute one of the most widely practiced training modalities, used both in competitive and recreational sports. However, excessive intensity of such training sessions provokes substantial muscle damage (rhabdomyolysis) and may lead to renal injury, which in severe cases is diagnosed as acute kidney injury (AKI). This necessitates hospitalization and renal replacement therapy, thereby affecting athletes’ health status and limiting their ability to participate in sports activities. The present work is a review of current knowledge on the phenomenon of rhabdomyolysis, its etiological factors, pathomechanisms, and health consequences, accompanied by a concise overview of emerging biomarkers of renal injury. The assessment of these biomarkers following physical exercise may provide new insights into the dynamics of post-exercise changes, indicate the severity and localization of exercise-induced renal damage, and contribute to a deeper understanding of structural kidney injury associated with strenuous physical activity—knowledge that may be applied in the prevention of exertional kidney injuries.

## Introduction

Fatigue after physical exercise serves two important functions. The first, and desirable one, is to stimulate adaptive processes based on changes resulting from microtraumas and disturbances in physiological/biochemical/molecular mechanisms. These processes constitute the physiological basis for functional and structural adaptations observed at various levels of the body's organisation (from molecular to tissue and organ changes). By this way, fatigue has a protective function—it defends the body against the overload and damage. On the other hand, if the right balance between work and rest is not maintained, and the effort (training) is dosed without sufficient knowledge, the body's adaptive mechanisms may become overloaded in various systems and organs, resulting in broadly understood overtraining. This predisposes the body to decreased physical performance, hormonal and vegetative changes, increased injury risk, as well as psychological and behavioural alterations (depression, aggression, irritability, conflict readiness) (Chung et al., 2021; Forys and Tokuhama-Espinosa, 2022; Toro-Roman et al. 2021).

In recent years, training regimes with very high intensity protocols have become increasingly popular in both competitive and amateur sports.

The desire to achieve quick results has led, particularly young people, often without prior physical preparation, to engage in very high-intensity exercise. Such risky behavior is usually accompanied by a lack of professional knowledge, which generates the risk of injuries and serious health consequences. Among very intense training workouts, the most popular ones currently are HIIT or CrossFit® (HIFT—high intensity functional training), strength training, blood flow restriction training (BFR/Kaatsu) and others. According to the *American College of Sport Medicine* (ACSM), high-intensity interval training (HIIT) was already a highly rated trend in the fitness industry in 2016 (Thompson, 2017), which persists to this day (Newsome et al., 2024). The variety of exercise forms, short intense phases of work and rest, and quick apparent training effects make it an ideal, economical and effective training method. Similarly, the combination of aerobic and resistance exercises in short, repetitive training sessions enhances the chances of developing strength, speed and endurance in a short period of time. This training strategy has become the basis for training individuals whose profession requires high physical fitness and great strength (police officers, special forces, firefighters) (Meier et al., 2023; Tabata et al., 2016).

CrossFit® integrates strength and fitness training (snatches, clean and jerks, squats, deadlifts, bench presses), gymnastics (pull-ups, sit-ups, lunges) and conditioning exercises (swimming, rowing, running). Since its introduction by Greg Glassman (Dawson, 2017), CrossFit® training has been quickly adopted by many military and civilian gyms. Currently, there are approximately 15,000 affiliated training centers and 15 million athletes worldwide (Meier et al., 2023). Although HIFT can be an enjoyable form of training and provide satisfaction, it is primarily a very intense form of exercise that often causes injuries and muscle damage (Weisenthal et al., 2014).

The high risk of injury does not deter those willing to participate in CrossFit® training. In addition to it, the high popularity of very intense training makes it important that emotionally engaged participants, who improve their physical fitness, cardiorespiratory fitness, strength and speed with a low training volume (Lee et al., 2018; Murawska-Cialowicz et al., 2015, 2021), are also aware of the adverse health consequences. By focusing on their goals and training too hard and exceeding the limits of their adaptive capabilities too quickly and unreasonably, thus failing to maintain a balance between work and rest, participants expose their bodies to high metabolic stress and disruption of homeostasis. However, introducing heavy loads into training too quickly can lead to an overload of both functional and structural adaptive mechanisms.

Overloading of the musculoskeletal system is a frequent topic of many scientific studies related to exercise, including very intense exercise (Plizga et al., 2024; Rynecki et al., 2019), but there is still a lack of reliable and large-scale studies on the overload of other organs, including the kidneys.

Literature devoted to physical exercise rarely addressed the kidneys and the relevance of the physiological phenomena regulated by them, even though proper kidney function has a direct impact on athletic performance and post-exercise recovery processes. Biochemical markers reporting the type and the degree of damage to the structure of the kidneys along with their physiological and health consequences are also rarely studied. Existing research in this area does not exhaust the problem and, considering intense exercise, there is a clear need to intensify and organize it, as there are occasional reports of the need for hospitalization of individuals after physical exercise and dialysis for amateur intense exercise practitioners who are unaware of the health consequences. The extreme clinical condition resulting from lack of preparation for intensive training and ignorance may lead to acute kidney injury (AKI).

In sports, we also encounter milder disorders leading to temporary functional impairment and damage to the structure of the kidneys, which directly impact the health, well-being and functioning of professional and amateur athletes. Limited functional efficiency of the kidneys determines the exercise capacity of athletes, the depth of fatigue changes and the speed of recovery and restoration of homeostasis disturbed by exercise. Thus, it can cause disturbances/overloading of physiological adaptation mechanisms and delay an athlete's return to form after intense physical work.

Therefore, for a better understanding of the very important issue of renal functional capacity related to both functional limitations and temporary damage to the delicate elements of the renal structure, a brief description of rhabdomyolysis—a phenomenon leading to kidney damage—is provided, along with an overview and description of selected biomarkers describing kidney function.

An additional objective was to identify which exercise-related situations most frequently triggered rhabdomyolysis and acute kidney injury, to describe the behavior of classical renal markers following intensive physical exertion, and to determine which emerging biomarkers show promise for the early detection of damage to renal structures.

To examine the issue of rhabdomyolysis in athletes and to present studies addressing biomarkers of kidney injury, a strategy of identification and selection was adopted based on the analysis of studies written in English and published in peer-reviewed journals accessible through the PubMed, Web of Science, Scopus, and SPORTDiscus databases. The processes of identification, review, and selection were conducted between September and December 2025 in order to include the most recent publications relevant to the research topic under discussion.

The selection of articles and scientific studies was based on the following keywords: “rhabdomyolysis”, “exercise”, “exercise-induced rhabdomyolysis”, “post-exercise kidney injury”, “AKI”, “high-intensity interval training”, “HIIT”, “CrossFit”, “high-intensity functional training”, “intensive training”, “renal function biomarkers”, and “kidney injury biomarkers”.

The search terms were combined using the operator “AND” to link keywords and “OR” to minimize duplication and maintain clarity of the search strategy.

## Kidneys

The kidneys are highly vascularised and innervated organs that maintain the balance of internal processes (homeostasis) and perform many functions that are essential for the body. The most important of these are: 1) excretory function: they act as a natural blood filter, removing water-soluble metabolic products and substances toxic to the body; 2) resorption: they reabsorb substances essential for the body (water, glucose, amino acids, ions, etc.); 3) maintaining water and electrolyte homeostasis by participating in the regulation of Na^+^, K^+^, Cl^-^, Ca^2+^, P0_4_^3-^ ion concentrations and acid-base balance, producing HCO_3_^-^ ions and regulating blood pH; 4) they perform an endocrine function (they are the site of synthesis of various hormones and biologically active substances, i.e., erythropoietin, renin, active forms of vitamin D_3_ and the site of action of hormones—vasopressin; 5) they participate in the regulation of blood pressure in the renin-angiotensin-aldosterone mechanism (Poortmans, 1984). They secrete irisin, which participates, among other things, in the regulation of inflammatory, energy and neuroprotective processes (Ciałowicz et al., 2025).

The basic structural and functional unit of the kidneys is the nephron, where the basic excretory processes of the kidneys take place: filtration in the glomerulus, reabsorption in the tubules, and production of primary and final urine. The preliminarily filtered plasma in the renal glomerulus enters Bowman's capsule, and from there, passes through Henle's loop and the distal tubules in the form of final urine, so metabolic waste products are excreted from the body while components present in primary urine that are essential for the body are reabsorbed in the distal tubules of the nephron. At rest, properly functioning kidneys act as a barrier that retains essential components, including proteins (Bertram et al., 2011). The filtration barrier consists of three layers within the glomerular blood vessel, including endothelial cells, the basement membrane and epithelial cells (mesangium and podocytes). The basement membrane is a layer of extracellular matrix (ECM) that forms a contact surface for endothelial and epithelial cells. The skeleton of the basement membrane is the result of the presence of collagen IV and laminin networks connected by nidogen, a structural protein fibronectin that is also present. The basement membrane is a cross-linked protein polymer, of which inner layer with the highest density determines the permeability of the filter, while the outer layers with a negative charge form an electrostatic barrier for plasma proteins, which in effect means that proteins with a molecular weight > 40 kDa are not filtered. The filtration properties of the basement membrane result from its high content of glycosaminoglycans (GAGs), mainly heparan sulphate and chondroitin sulphate (Madrazo-Ibarra and Vaitla, 2023). By binding to extracellular matrix proteins, glycosaminoglycans impart a negative charge, which prevents even proteins with lower molecular weights from passing through the pores in the basement membrane. Both laminin and fibronectin, as extracellular matrix proteins, have the ability to bind to collagen IV and GAGs, including heparan sulphate (Schmidt et al., 2016).

Collagen IV is the main structural component of every basement membrane. It provides a scaffold for epithelial and endothelial cells in tissues. In the glomeruli, it is the main component of the layer known as the *lamina densa*. In this layer, it is responsible for counteracting mechanical forces and determines the stability of nephron structures (Quinlan and Rheault, 2021). It is characterised by a specific four-domain structure of polypeptide chains and a specific amino acid sequence, which increases the sensitivity of this protein to proteolytic enzymes. Membrane proteases play an important role in the modulation and functioning of basement membranes (e.g., they enable immune system cells, leukocytes, to pass through the nephron filtration barrier). Enzymes that catalyse the proteolysis of proteins in the remodelling and degradation of the extracellular matrix include metalloproteinases (MMPs) of the collagenase, gelatinase and elastase groups. Metalloproteinase 9 (MMP-9) belongs to the gelatinase group, which degrades, among others, type IV collagen and fibronectin. Increased activity of this enzyme (caused by the interaction of pH changes, cytokines, interleukins and others) can cause degradation of the structures of the nephron basement membrane responsible for the selective permeability of proteins in the renal glomerulus.

Under resting conditions in healthy individuals, urine, which is a filtrate of blood, has a relatively constant biological and physicochemical composition, and the biological substances it contains originate from plasma filtered through the glomerular filtration membrane or secreted by renal cells (Bakońska-Pacoń and Borkowski, 2003).

## Kidneys and Physical Exercise

### Blood Flow through the Kidneys during Intense Exercise

Under resting homeostasis conditions the blood flow through the kidneys is 20%–25% of cardiac output (approx. 1200 ml/min) (Czarkowska-Pączek et al., 2020), and the volume of plasma filtered by the glomeruli reaching Bowman's capsule in one minute determines the glomerular filtration rate (GFR). The higher the GFR, the more efficient the filtration and the faster the removal of waste products. In healthy adults, this value should be > 90ml/min/1.73 m^2^ (usually around 120–130 ml/min/1.73 m^2^), which is approximately 10% of the blood flowing through the kidneys in one minute.

Physical exercise causes profound changes in renal haemodynamics and the excretion of metabolites and proteins. It causes a decrease in the renal blood flow (RBF) proportional to the effort intensity, which can be significantly reduced, even by 25% compared to the RBF at rest, and renal vascular resistance can increase up to 5-fold (Poortmans, 1984). The GFR also decreases during exercise. It has been demonstrated that these indicators depend more on the intensity than on the duration of exercise. However, at low and moderate exercise intensities, no changes in the GFR are observed (Poortmans, 1984), which is why light and moderate exercise is currently recommended in the rehabilitation process of patients with chronic kidney disease (CKD) (Ciałowicz et al., 2025). According to research, a GFR below 30 ml/min/1.73 m^2^ may indicate severe renal failure (Rout and Aslam, 2025).

Similarly, creatinine clearance gradually decreases only at an exercise intensity of approximately 40–60% VO_2max_. The studies by Suzuki et al. (1996) and Kawakami et al. (2018) support that creatinine clearance decreases rapidly by almost ½ when the exercise intensity is above 80% VO_2max_. Creatinine clearance is a classic biomarker and measure of renal filtration function. This clearance determines the volume of plasma that the kidneys will clear of creatinine in one minute. Currently, it is one of the traditional indicators for assessing glomerular filtration and the ability of the kidneys to remove metabolic waste products, and is used to detect and monitor renal failure (Bongers et al., 2018). Unfortunately, the diagnostic value of this variable is significantly limited in cases of oliguria or anuria, which may occur after very intense exercise (Kumar et al., 2022).

The study by Suzuki et al. (1996) additionally reported a significant reduction of ~46% in the renal plasma flow (RPF) during recovery after exhaustive exercise on a bicycle ergometer compared to the resting value, and after 30 and 60 min of recovery to 82.5% and 79%, respectively. Such changes in the blood and plasma flow through the kidneys significantly reduce the removal of metabolic products from the body, which has an impact on the functioning of the body. The RPF determines the actual volume of plasma that flows through the kidneys and reaches the glomeruli in one minute, thus it indicates how much plasma is filtered by the glomeruli and is a key indicator of kidney function. Those authors believed that the cause of these changes was an increased sympathetic innervation of the kidneys and increased catecholamine secretion during exercise and recovery (Suzuki et al., 1996). The renin-angiotensin-aldosterone system (RAA) also plays a regulatory role in the renal blood flow (Poortmans et al., 2001). Variables such as the RBF, RPF, GFR, and the urine flow rate are reduced during intense exercise because the body's physiological adaptation to exercise caused by increased activity of the renal sympathetic nerve (Bardgett et al., 2014; Montero, 2019), the release of vasopressin, and the activation of the renin-angiotensin-aldosterone system promote fluid conservation and protect the body from fluid loss and dehydration in the short term due to water and electrolyte loss through sweating (Montero, 2019; Schlader et al., 2019).

Unlike physical exercise above the threshold intensity, haemodynamic variables reflecting kidney function do not decrease during exercise below the lactate threshold, i.e., below 35–40% VO_2max_ (Suzuki, 2015). According to Fukuta et al. (2024), in healthy individuals, the RBF below the ventilatory threshold does not differ from the resting value, but decreases significantly after exceeding this threshold.

During exercise, the blood flow through the kidneys is significantly reduced as due to the increased oxygen demands the blood flow is directed to the skeletal muscles, which under these conditions use approximately 80% of cardiac output (Joyner and Casey, 2015). This adaptive mechanism, at very high exercise intensities, can promote renal hypoxia, affect the ATP/ADP ratio, provoke oxidative stress, disrupt basic renal regulatory functions, and cause damage to renal structures. It is believed that due to the specific nature of blood circulation within the kidneys and reduced oxygen availability, the kidneys are highly susceptible to ischaemic damage (Brezis and Rosen, 1995). The damage may be exacerbated by a number of internal changes and external environmental factors. In addition, excessive exercise intensity can cause skeletal muscle damage (rhabdomyolysis), which is the most recognisable factor causing kidney damage in sports. It is believed that ischaemic acute kidney injury resulting from insufficient blood supply accounts for approximately 50% of cases of critically ill patients with AKI (Almulhim, 2025). As a result of haemodynamic adaptive changes during exercise, increased blood pressure and a decreased renal blood flow, there are significant disturbances in glomerular filtration and reabsorption, which also has a direct impact on the composition of urine after exercise. There is also intensity-dependent damage to the structure of various nephron elements, the appearance of which in the urine may indicate both the location and extent of the damage (Suzuki, 2015).

### Post-Exercise Proteinuria

According to Poortmans (1984, 1985), the protein profile in urine (proteinuria) after exercise changes according to the intensity of the exercise, where after intense exercise, glomerular-tubular proteinuria predominates, while after low-intensity exercise, it is usually glomerular proteinuria that predominates. This effect of exercise intensity on clearance of individual plasma proteins suggests an increase in glomerular permeability and/or a partial inhibition of tubular reabsorption of macromolecules.

Many researchers investigate the phenomenon of post-exercise proteinuria, but these studies mainly concern the variables of tubular disorders by determining changes in low molecular weight protein concentrations, while there are only few reports on changes in the nephron basement membrane. A significant increase in protein concentration in post-exercise urine indicates high intensity physical activity. According to Poortmans et al. (2001), these changes are associated with high concentrations of lactic acid and catecholamines in the blood, which in turn leads, among other things, to changes in the permeability of the glomerular basement membrane and renal tubules, as well as changes in the blood flow in the kidney (Wołyniec et al., 2020). Post-exercise proteinuria indicates impaired permeability of the glomerular basement membrane as a result of the reduced blood flow in the kidneys, increasing the diffusion of proteins into the tubule lumen. High-molecular-weight proteins appear mainly in prolonged submaximal exercise. Poortmans (1985) demonstrated that running a distance of 300 m caused a tenfold increase in total protein concentration in post-exercise urine, a twenty-seven-fold increase in albumin concentration, and a threefold increase in low molecular weight protein concentrations, i.e., α_1_-microglobulin and β_2_-microglobulin. A very large increase in total protein concentration (4600%) was also found in the urine of triathletes after exercise, and only a 3-fold increase in β_2_-microglobulin concentration (Yaguchi et al., 1998).

Short-term exercise causes disturbances in reabsorption in the renal tubules (tubular proteinuria), resulting in an increased passage of low molecular weight proteins such as β_2_-microglobulin, lysozyme and retinol-binding protein (RBP) into the urine (Neumayr et al., 2003; Poortmans et al., 1997; Touchberry et al., 2004). In a sample of 400-m runners of the Poortmans et al.'s (1997) study, the concentration of total protein in post-exercise urine increased 30-fold, albumin 10-fold, α_1_-microglobulin 12-fold, and β_2_-microglobulin 20-fold.

In addition to total protein levels in urine, the activity of selected enzymes can be used to monitor training (Bakońska-Pacoń and Borkowski, 2003). One of the enzymes used in assessing kidney function is N-acetyl-ß-D-glycosaminidase (NAG), a lysosomal enzyme found in the proximal tubules of the nephron, which is a recognized marker of early renal changes (Bakońska-Pacoń and Borkowski, 2003; Bosomworth et al., 1999). During intense exercise, the antidiuretic hormone (vasopressin) also plays a significant role, as the filtration rate depends on the degree of hydration of the body. Heavy physical work also leads to increased excretion of erythrocytes and leukocytes in the urine. Protein excretion in the urine with an intact filtration barrier is a temporary condition and may persist for up to 2 h after exercise (Poortmans and Vanderstraeten, 1994), although some studies indicate longer persistence of proteinuria. As a result of prolonged intense exercise, haemoglobinuria and the aforementioned myoglobinuria are also observed, and indicate serious kidney damage (Suzuki, 2015; Suzuki et al., 1996).

## Rhabdomyolysis

### A Brief Historical Overview

Rhabdomyolysis is not a rare disease, given the variety of factors that cause it. It was already described in ancient times as a condition called *coturnism*, caused by the consumption of quails (*Coturnix cotunix*) that fed on various plants that were toxic to humans. In contemporary descriptions, rhabdomyolysis functions as a ‘crash syndrome’ and has been described mainly in the context of disasters (e.g., in victims of the 1908 Sicilian earthquake or the 1941 London air raids). A broader historical overview of the occurrence of this phenomenon in various situations has been provided by various authors (Aleckovic-Halilovic et al., 2020; Rout et al., 2025). Rhabdomyolysis remains an important topic in military and disaster medicine. However, it seems that its severity in the context of exercise has never been sufficiently appreciated and has not been given sufficient attention. Recent studies indicate that the incidence of rhabdomyolysis among military personnel was 22.2 cases per 100,000 exercisers per year (Alpers and Jones Jr., 2010).

### Characteristics of the Phenomenon in the Context of Exercise

Rhabdomyolysis is most often triggered by intense or prolonged exercise. It is more common in people who are not adapted to exercise (Rawson et al., 2017). Its occurrence is facilitated by external conditions, especially high ambient temperature and humidity, as well as internal conditions such as hyperthermia, dehydration or electrolyte disturbances (Junglee et al., 2013).

Rhabdomyolysis can be defined as a clinical entity in which skeletal muscle damage occurs with the release of muscle fibre contents, including ions, enzymes and proteins, into the bloodstream. Depending on its intensity, rhabdomyolysis can take various forms and manifest itself in different ways—from an asymptomatic form with elevated creatine kinase (CK) levels as for example in the study of Podgórski et al. (2021) to a severe clinical condition with extremely elevated CK levels, electrolyte disturbances, acute renal failure or DIC syndrome (disseminated intravascular coagulation, consisting of generalised activation of the blood clotting process, combined with activation or inhibition of fibrinolysis).

Rhabdomyolysis is accompanied by a set of symptoms, i.e., severe muscle pain (myalgia), swelling and weakness of muscles, dark urine sometimes resembling the colour of tea or coca-cola. However, according to Torres et al. (2015), this triad of symptoms can be misleading, as in clinical studies less than 10% of people who have developed rhabdomyolysis have all three symptoms, and >50% of patients do not complain of muscle pain or weakness. Oliguria or anuria is also observed during rhabdomyolysis (Torres et al., 2015). The set of symptoms that make up rhabdomyolysis leads to systemic complications which may result in a potentially life-threatening condition (Sauret et al., 2002).

An example of a mild form of rhabdomyolysis is *Delayed Onset Muscle Soreness* (DOMS), which occurs several to several dozen hours after intense or unusual exercise. This set of pain symptoms can last up to several days. When it manifests as extensive muscle damage, it becomes a significant clinical problem. Exercise-induced rhabdomyolysis (EIR) can lead to various complications classified as early or late. Early complications include severe hyperkalaemia, which promotes cardiac arrhythmias and can lead to cardiac arrest (Cortesi et al., 2010). To avoid undesirable effects, athletes often attempt to reduce acidification of the body and improve their ability to perform intense exercise. To this end, they ingest bicarbonate-rich water combined with an alkalizing diet which affects the acid-base balance and anaerobic performance (Chiron et al., 2024).

### Causes of Exercise Induced Rhabdomyolysis

There are numerous causes of rhabdomyolysis (Bäcker et al., 2023). In the field of sports, they mainly include injuries, muscle damage, compression, eccentric contraction exercises, exercises that provoke ischaemia (Kaatsu Training/Occlusion Training/Blood Flow Restriction (BFR)) (Iversen and Røstad, 2010; Tabata et al., 2016), weightlifting, marathons, military training, excessive exertion (Iversen and Røstad, 2010; Tabata et al., 2016), as well as low physical fitness, premature introduction of repetitive eccentric exercises (squats, push-ups and sit-ups) (Pierson et al., 2014), and electrolyte disturbances (e.g., hypokalaemia, hyponatraemia).

Eccentric exercises are part of various high-intensity training programmes such as CrossFit®, HIIT, Insanity, Gym Jones or Tabata. These programmes involve intense exercise with short rest intervals. In endurance activities, such as marathon running, rhabdomyolysis is additionally provoked by other factors, including environmental ones (Clarkson, 2007). As mentioned, exercise-induced rhabdomyolysis mainly affects untrained individuals who exceed their exercise capacity, especially on hot days. Individuals who are adapted to exercise may experience rhabdomyolysis when they increase their training regime requiring intense eccentric contractions, increase its volume or intensity. It can occur also during training camps in the pre-season period or after a long break associated with a reduced level of training (Scalco et al., 2016). Eichner (2010) believes that rhabdomyolysis can occur in anyone who does ‘too much, too fast, too soon, too new’ exercise. Other factors that can exacerbate post-exercise rhabdomyolysis include various medications, such as statins and non-steroidal anti-inflammatory drugs (NSAIDs) which are often used by athletes, as well as internal endocrine and metabolic factors (e.g., diabetic ketoacidosis, lactic acidosis, hypothyroidism), sickle cell anaemia, toxins of various origins or infections, and genetic disorders—muscular dystrophies (Rawson et al., 2017; Sauret et al., 2002; Zutt et al., 2014a). In the context of exercise, genetic factors that exacerbate rhabdomyolysis include genetic disorders such as McArdle's disease (deficiency of glycogen phosphorylase in muscles), Tarui's disease (deficiency of phosphofructokinase in skeletal muscle cells), and lipid myopathies, including carnitine palmitoyltransferase (CPT I and II) deficiency (Durka-Kęsy et al., 2012).

Other genetic disorders include Krebs cycle disorders, mitochondrial respiratory chain disorders, glucose-6-phosphate dehydrogenase deficiency phosphate dehydrogenase (G6PDH) deficiency, and myoadenylate deaminase (MAD) deficiency, an enzyme responsible for energy production and the conversion of AMP to IMP. Its deficiency leads to symptoms similar to exercise-induced pain (Baumeister et al., 1993; Torres et al., 2015; Yang et al., 2025), thiolase deficiency resulting in β-oxidation disorders, increased susceptibility to malignant hyperthermia, type 1 ryanodine receptor (*RyR1*) mutations (Scalco et al., 2016), including single polymorphisms of the *CKMM Nco l, ACTN3 R577X* and *MYLK C37885A* genes may also be associated with more severe symptoms of post-exercise rhabdomyolysis (Deuster et al., 2013; Scalco et al., 2016).

It is important to remember that we are all different, and when undertaking physical activity, we must be aware of and accept our limitations and know our physical capabilities (Sunder et al., 2019).

**Figure 1:**
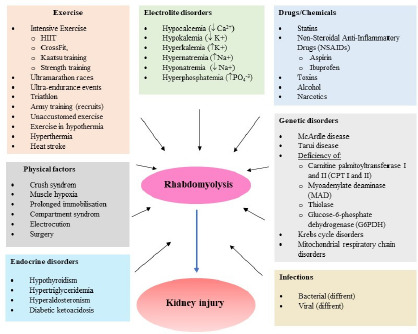
Selected factors causing rhabdomyolysis.

Alcohol, drugs (Chandrasekhar et al., 2024) and other stimulants are also contributing factors (Larbi, 1998; Rawson et al., 2017; Zutt et al., 2014a). Due to the percentage of muscle mass, exercise induced rhabdomyolysis is more common in men, African Americans, people who are not adapted to exercise, who are excessively obese, and who chronically use lipid-lowering drugs (statins) and anti-inflammatory drugs. The factors causing rhabdomyolysis are described in more detail in other papers (Aleckovic-Halilovic et al., 2020; Newsome et al., 2024; Torres et al., 2015; Yang et al., 2025), and Figure 1 presents them in a synthetic manner.

When combined with various complications, rhabdomyolysis can have very serious health consequences. Therefore, it is important to be aware of its occurrence and the health risks induced by physical exertion. Rapid diagnosis and intervention at an early stage of skeletal muscle damage are extremely helpful in preventing complications and ensuring successful treatment. The basic form of therapy is to administer intravenous infusions of physiological fluids with calcium bicarbonate as soon as possible to remove myoglobin from the kidneys, restore diuresis and raise the pH of the urine. It is also important to be aware of one's physical limitations and to gradually increase training loads, rather than suddenly undertake strenuous exercise (Tabata et al., 2016).

If muscle damage occurs with the release of muscle proteins into the blood, including myoglobin in high concentrations and under favourable conditions (e.g., dehydration or heat stress), myoglobin accumulates in the kidneys, causing acute renal failure/acute kidney injury (AKI) (Clarkson, 2007).

Under physiological conditions, myoglobin is easily filtered by the glomerulus and quickly passes from the blood into the urine. In large quantities, it poses a health and therapeutic problem because, when it enters the lumen of the renal tubules, it reacts with Tamm-Horsfall protein (THP, uromodulin) and precipitates, especially in acidic urine, causing obstruction of the distal tubules. Tamm-Horsfall protein is secreted by the distal tubules of the nephron and is one of the components of casts in urine sediment. Myoglobinuria (tea-coloured urine, coca-cola-coloured urine) occurs when the concentration of myoglobin in the urine exceeds 250 µg/ml (normal <5 ng/ml). Oxidative stress accompanying muscle and kidney cell damage promotes the oxidation of Fe^2+^ to Fe^3+.^ The resulting OH∙ radical in the presence of haem iron may further contribute to toxic damage to the tubules. The precipitation of myoglobin is counteracted by both higher urine alkalinity and urine volume (Scalco et al., 2016).

In addition to acute renal failure and damage, exercise-induced rhabdomyolysis also leads to complications such as heart failure, arrhythmia, electrolyte imbalance, liver dysfunction, disseminated intravascular coagulation (DIC), muscle oedema, and therefore fascial compartment syndrome, and in severe cases even death (Petejova and Martinek, 2014). Therefore, in order to prevent this condition, it is important to be aware of one of the basic laws of training—gradually increasing the intensity and duration of exercise, as opposed to sudden and reckless intense effort (Sunder et al., 2019). One case involving an obese man who underwent BFR training for the first time in his life indicates that public awareness of training is not sufficiently developed and that intensive training should be conducted under the supervision of a personal trainer, taking into account the physical condition and strength capabilities of the individual in order to prevent serious complications (Tabata et al., 2016).

Given the unpredictable health consequences, one of the primary goals in the treatment of exercise rhabdomyolysis is the prevention of acute kidney injury, which is diagnosed—aside from the concentration of myoglobin in blood and urine—on the basis of urine output (as intensive physical exertion may lead to oliguria or anuria), serum creatinine levels, electrolyte concentrations, and additionally, through the assessment of liver enzyme activity aspartate aminotransferase (AST), alanine aminotransferase (ALT), lactate dehydrogenase (LDH), but most importantly through the measurement of the rhabdomyolysis marker considered the gold standard, namely CK, of which reference ranges vary across research centers, typically falling within 200–300 U/l (Gur and Simsek, 2024; Kumar et al., 2022). The activity of this enzyme is widely recognized as a predictive factor for the likelihood of acute renal failure, with levels exceeding 5000 U/l strongly associated with the development of renal damage (Cervellin et al., 2010; Rout et al., 2025). Although there is currently no universally accepted diagnostic criterion for rhabdomyolysis, numerous studies and hospital practices adopt a fivefold increase in CK activity above the upper limit of normal, i.e., above 1000 U/l, as the diagnostic threshold (Cervellin et al., 2010), with 1500 U/l being the value most commonly applied (Gur and Simsek, 2024; Torres et al., 2015), though some authors consider this criterion too rigorous. George et al. (2010) argue that less than a tenfold increase (CK <3000 U/l) represents a low level of enzyme elevation. Those authors also suggest that in individuals with mild CK elevation (<15,000 U/l), normal creatinine concentrations, and in the absence of additional risk factors such as dehydration, sickle cell anemia, use of analgesics, infections, or metabolic syndrome, the risk of complications following exertional rhabdomyolysis is low.

As a consequence of skeletal muscle injury, creatine kinase activity begins to rise approximately 2–12 h after the insult, reaching peak values within 24–72 h. The half-life of CK is 1.5 days and it persists longer than myoglobin, of which half-life is 2–4 h and returns to normal within 6–8 h after muscle injury (Rout et al., 2025; Scalco et al., 2016). Due to its short half-life, serum myoglobin concentration is unfortunately less diagnostically sensitive than CK, which may lead to misinterpretation and false-negative test results. Urine dipstick testing may yield a positive result for the presence of erythrocytes, as ortho-tolidine on the test strip turns blue in the presence of myoglobin (Torres et al., 2015). According to Gur and Simsek (2024), an important marker in assessing injury severity and health consequences is also the analysis of base excess (BE), determined by blood pH and HCO_3_⁻ concentration. BE is a marker of metabolic acidosis, which is exacerbated by impaired tissue perfusion, and may be useful in predicting the severity of injury as well as associated clinical outcomes (Davis et al., 2020). Selected cases of rhabdomyolysis after intense exercise in athletes or amateurs with serious health consequences are presented in Table 1a and Table 1b.

**Table 1a:** Selected scientific publications describing cases of rhabdomyolysis, kidney damage and therapeutic measures after intense physical exercise.

Ref.	Type of exercise/Event	Diagnosis	Symptoms	Blood test results	Urine test results	Follow up/Recovery time
(Morton and Waldek, 1985)	♂ Master in Tae Kwon Do / Breaking of 2035 boards / 4 h	Acute kidney injury	Back pain	Myoglobinemia CK = 1600 IU/L	Myoglobin in urine, blood in urine (strip test), no RBC	Return for function after four hemodialysis sessions
(Tabata et al., 2016)	♂ 31 years, obese / BFR training session and resistance exercises	Acute EIR based on CK; acute tonsillitis	Next morning severe muscles’ pain of lower and upper limbs, a high fever (39.9°C)	Admission: CK = 56 475 U/L 2^nd^ day: peak CK = 89 824 U/L WBC = 17 390/μl CRP = 10.43 mg/dl ↑↑ ALT, AST, LDH	Myoglobin >3000 ng/ml	Aggressive intravenous (IV) infusions of NaHCO_3_; on the 10^th^ day the patient was discharged
(Sauret and Jaen, 2000)	♂ 19 years / vigorous workout (vertical jumps, running, leg presses)	Pain in both legs; brown color of the urine	2 days before training cold-like symptoms; evening before admission the patient drank 10 beers	Admission: CK = 23 1700 U/L ALT = 490 U/L AST = 2560 U/L LDH = 3450 U/L	Dark-coloured urine; blood in urine (strip test); Myoglobin = 2500 ng/ml	IV infusion of 1 mL of NaHCO_3_/L of 0.9% NaCl to alkalinize the urine
(Sunder et al., 2019)	♂ 35 years / strenuous workout followed by fasting for one day	Dark urine, EIR, AKI	Overall weakness, decreased urine output; AKI with a frank renal shutdown	Admission: CK = 2937.7 IU/ml GOT = 2332.1 IU/ml GPT = 642.9 IU/ml Cr =12.2 mg/dl Urea = 148 mg/dl PO_4_^3-^ = 5.8 4 mg/dl HCO_3−_ = 17 mEq/l Na^+^ = 122 mEq/l		Anuria for 24 h; 7 sessions of haemodialysis each lasting 4 h but with poor ultrafiltration (50 mL). After the fifth dialysis the urine volume was larger. The patient was discharged after about a month
(Honda et al., 2017)	♂ 37 years / previously healthy; BMI: 30.8 kg/m^2^/ HIRT/100 pushups, 100 exercises using a 20-kg dumbbell, 50 lifts using a 10-kg dumbbell	EIR; no previous experience in such intensive training; the patient was taking loxoprofen sodium	A day after training he noticed dark urine and several myalgia. After few days myalgia was gradually alleviated, but the dark urine continued	Admission: CK = 95 100 U/L LDH = 4750 U/l AST = 999 U/l ALT = 443 U/l CRP = 1.9 mg/dl Cr = 1.05 mg/dl	Light brown urine; blood and protein in urine; Myoglobin = 160 000 ng/mL	eGFR, mL/min/1.73 m^2^ ≥60, myalgia gradually subsided, as did the CK level. The patient was discharged after 9 days. Two weeks after discharge, CK remained mildly elevated at 280 U/L
(Meyer et al., 2018)	♀ 31 years / CrossFit® workout: variety of exercises (pushups, plyometrics, weightlifting)	EIR	2 days after training, biceps pain and soreness; upper extremity swelling	Admission: CK = 18 441 U/L AST = 462 U/L ALT = 155 U/L	Urine not darkened	Renal function was normal. The patient was hospitalized for 2 days and treated with intravenous fluids
(Reyes et al., 2022)	♂ 32 years / very intensive CrossFit®; 2 days earlier with intense work on lower limbs which he was not familiar with	EIR with AKI	Pain of abdominal, lumbar and thigh regions; changes in color of urine	Admission: CK = 189 000 U/L GRF = 57.9 ml/min/1.73m^2^ AST = 3185 U/L ALT = 589 U/L	RBC and protein in urine	Hydration with IV fluids for a target diuresis within 200–300 ml/h; no need for renal support therapy in the ICU. In 7 days, parameters decreased to lower values
(Busanello Mata Alves et al., 2024)	♂ 45 years / Level 2 CrossFit® Coach (CF-L2) performed a routine CrossFit® workout	EIR	2 days after training muscle stiffening and progressive pain of upper limbs upon palpation; dark urine	Admission: CK = 126 891 U/L AST = 2154 U/L, ALT = 568 U/L, LDH = 4067 U/L	Brown urine Hb (+++) pH = 6.5	Treated with aggressive IV and oral hydration; dismissed after the fifth day of hospitalization, without any further complications

**Table 1b:** Selected scientific publications describing cases of rhabdomyolysis, kidney damage and therapeutic measures after intense physical exercise.

(Lawrensia et al., 2021)	♂ 27 years, healthy and physically active / CrossFit®: first training after not doing any exercise or a CrossFit® session for three months	EIR, no analgesics to alleviate pain	Urine tea-colored; soreness in both lower extremity muscles, which caused difficulty walking	Admission: CK = 54 240 U/L Ca^2+^ = 1.87 mmol/L LDH = 1670 U/L Cr = 70 mmol/L	Blood and RBC present in urine	Treated with IV isotonic solution up to 2 L/24 h and analgesic agents. The patient drunk water (5–6 L/day). Urine output was at 2.4 mL/kg/h. On the third day, the patient was discharged, His urine color was normal.
(Adhikari et al., 2021)	♂ 22 years / one exhaustive CrossFit® session.He did not report using statin, dietary supplements, NSAIDs or alcohol	EIR	Two days after training, the patient sustained myalgia/severe pain around the chest, the upper back and shoulders	Admission: CK = 132 540 U/L AST = 136 U/L ALT = 772 U/L Cr = 1.05 mg/dL	Dark-coloured urine; blood in urine; RBC not present	Treated with aggressive IV fluids and oral hydration therapy. The patient did not develop any complication and was discharged on the sixth day
(Tibana et al., 2018)	♀ 35 years / extreme conditioning program (ECP) competition composed of five workouts performed on three consecutive days	EIR, pain and soreness; myalgia up to 25 days after the ECP competition	Pain began one day after ECP competition	Admission: CK = 43 322 U/L Myoglobin = 1350 ng/mL AST = 477 U/L ALT = 74 U/L	Renal function normal	Discharged on the fourth day of hospitalization
(Clark and Manini, 2017)	♂ 20 years / performed 6 sets of blood KAATSU exercise (3 sets of knee-extension and 3 sets of elbow-flexion exercise)	EIR	High levels of delayed onset muscle soreness in the days after the exercise bout	Admission: CK = 36 000 IU/L		Hospitalized for exertional rhabdomyolysis
(Al Badi et al., 2020)	♂ 19 years / intense aerobic exercise	EIR with AKI and DIC	Bilateral leg pain, dark urine	Admission: CK = 587 600 IU Hb = 18.5 g/dl WBC = 33 200/µl LA = 6.5 mmol/l ALT = 2561 U/l AST = 6632 U/l	Urine blood (+++) pH = 6.0 Urine protein (trace)	Double fasciotomy to relieve compartment syndrome; several sessions of hemodialysis. The patient’s condition was complicated by bacteriemia and resistance (MDR). The patient was discharged after 3 weeks with a follow-up physiotherapy because of the bilateral foot drop
(Kumar et al., 2022)	♂ 26 years / heavy exercise without supervision	Three days after EIR and AKI	Feeling fatigued; brown urine; decreased urine output on the next day; bilateral posterior thigh and calf pain; the patient could not bend on his knees	Admission: CK = 87 750 U/L Cr = 12.65 mg/dl Urea = 201 mg/dl LDH = 787 U/L Na^+^ = 126 mEq/l Ca^2+^ = 6.69 mg/l PO_4_^3-^ = 6.94 mg/l Metabolic acidosis HCO_3_^−^ = 15 mEql/l pCO_2_ = 24.1 mmHg	Urine analysis negative for protein and occult blood	One day after admission, a higher level of creatinine and urea than on day 1. No improvements following intravenous hydrotherapy—no significant urinary output for 24 h. Hemodialysis on the 2^nd^ day of admission. A total of 5 hemodialysis sessions were done. The patient was discharged on the 6^th^ day of hospital admission

BFR: blood flow restriction; AKI: acute kidney injury; EIR: exercise-induced rhabdomyolysis; NSAIDs: non-steroidal anti-inflammatory drugs; CK: creatinine kinase; CRP-C: reactive protein; ALT: alanine transaminase; AST: aspartate transaminase; LDH: lactate dehydrogenase; GOT (AST): glutamic-oxaloacetic transaminase; GPT (ALT): glutamic pyruvic transferase; Cr: creatinine; RBC: red blood cells; HIRT: high intensity resistance training; DIC: disseminated intravascular coagulation; IV: intravenous; BMI: body mass index; GFR: glomerular filtration rate; ICU: Intensive Care Unit

Studies conducted between 2009 and 2013 among US Army soldiers showed that during the study period, the incidence of exercise induced rhabdomyolysis was approximately 33%. The highest incidence rate was observed in male recruits under the age of 20, of Asian/Pacific or African Americans, not of Latin American origin, soldiers in the Army or Marine Corps and individuals performing combat-related professions (Armed Forces Health Surveillance Center (AFHSC), 2014). In 2016, there were 525 cases of exercise-induced rhabdomyolysis provoked by intense military training and heat stress. Studies conducted between 2020 and 2024 showed slightly lower but stable incidence rates per 100,000 (38.0 to 38.4 cases per 100,000). The average hospitalisation rate was 38.8%. Exercise induced rhabdomyolysis was most frequently recorded in recruit training bases, with the highest incidence in the months from May to August.

These data only confirm existing knowledge and point to the fact that training is much more intensive in the types of combat units presented than in other military groups, and that recruits may be exposed to environmental conditions requiring acclimatization to high temperatures and humidity during the warmer months, as soldiers and marines in combat units often undergo very rigorous physical training, fitness training and field exercises, regardless of weather conditions (Update, 2025). Other authors (Chavez et al., 2016) also confirm that rhabdomyolysis is more common in males, obese individuals, especially those with a BMI exceeding 40 kg/m^2^.

The knowledge of coaches and athletes about the existing health threat and mortal risk of this condition is a requirement of modern times, in the era of post-pandemic, rapid development of fitness services, individuals exercising in gyms without supervision and in ‘home gyms’, as well as in the context of mass events, e.g., marathons, half marathons, extreme ultramarathons (e.g., Badwater Ultramarathon), Ironman competitions or popular ‘charity’ or ‘special occasion’ runs, which are guided by a noble cause. It may be the first and most important link in saving a life (Tidmas et al., 2022). It happens that during mass runs, competitors faint. There have also been fatalities (Dayer and Green, 2019) which might be caused, among other things, by a lack of adequate athletic preparation, recklessness resulting from ignorance and unfamiliarity with the condition of one's body. Knowledge of the consequences of strenuous exercise under difficult environmental conditions (temperature, humidity, sunlight), the physiological reactions that occur in the body during intense physical activity, and rapid intervention and treatment of complications ensure recovery and are the most important measures in post-exercise rhabdomyolysis (Rubio-Arias et al., 2019; Scheer et al., 2022). Meanwhile, as research shows, ultra-endurance events, defined by distance (>42.195 km for running), duration (>6 h or several days/stages) and environment (mountains, desert), are becoming increasingly popular (Lecina et al., 2021; Rojas-Valverde et al., 2021; Rubio-Arias et al., 2019; Scheer et al., 2022).

According to Tidmas et al. (2022), acute kidney injury and other health-damaging pathologies associated with ischaemic damage often develop in ultra-endurance athletes due to the severity of the effort, the high level of exposure to functional and structural damage, and the number of NSAIDs taken, hyponatraemia, and nitrate supplementation. Tidmans et al. (2022) also reviewed 22 studies in which other authors presented a number of individuals and health effects, including kidney damage, in athletes competing in various types of ultramarathons. After analysing 22 articles describing the occurrence of various health consequences, including post-exercise rhabdomyolysis and AKI, it was observed that out of 1,059 athletes participating in ultra-marathon races, 445 athletes met the criteria for AKI from the risk group, and 281 met the criteria for AKI related to injuries.

Those studies only confirm that there is a high health risk after intense exercise and, therefore, it is worth educating everyone involved in sports, from participants to coaching staff and doctors. An interesting review of the literature on rhabdomyolysis and the incidence of acute kidney injury in athletes was conducted by Rojas-Valverde et al. (2021). A similarly interesting predictive model for AKI occurrence in 24-hour ultramarathon runners was proposed by Hsu et al. (2022).

Sunder et al. (2019) believe that the clinical symptoms of rhabdomyolysis may not be present initially, which can be misinterpreted and delayed therapeutic intervention may cause serious complication. Many patients may develop acute renal failure requiring dialysis. As it was mentioned before, the occurrence of acute kidney injury is also often associated with alcohol abuse, drug abuse or non-steroidal anti-inflammatory drugs (NSAIDs) which are often taken by athletes (Deighan et al., 2000).

### Pathomechanism of Rhabdomyolysis

The pathomechanism of rhabdomyolysis is based on damage to the cell membrane of muscle fibres and an increase in intracellular calcium concentration. This causes excessive fibre contractility, protease activation, induces oxidative stress and the production of reactive oxygen species (ROS), mitochondrial destruction and a decrease in energy production, leading to cell death (Kim et al., 2016; Torres et al., 2015). Under physiological conditions, calcium homeostasis in skeletal muscle cells is maintained by ATP-dependent cell membrane proteins (channels, pumps), including Na^+^/K^+^ ATPase and Ca^2+^ ATPase. Under physiological conditions, they maintain low intracellular concentrations of Na^+^ and Ca^2+^ and high intracellular concentrations of K^+^. In the event of energy deficiency, membrane proteins become dysfunctional. This results in increased membrane permeability to Na^+^ and Ca^2+^ ions. The 2Na^+^/Ca^2+^ exchanger increases the intracellular concentration of calcium. Ca^2+^ ATPase is unable to pump out excess of intracellular calcium due to energy depletion. The secondary increase in intracellular Ca^2+^ rises the activation of calcium-dependent proteases and phospholipases, including phospholipase A2 (PLA2), which destroy the structure of the cell membrane and the mitochondrial membrane, as well as myofibrillar proteins and cytoskeletal proteins.

**Figure 2:**
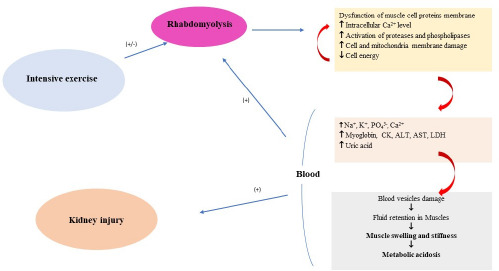
Simplified pathomechanism of rhabdomyolysis. CK: creatinine kinase; ALT: alanine transaminase; AST: aspartate transaminase; LDH: lactate dehydrogenase

The intracellular excess of Ca^2+^ also disrupts mitochondrial structure and induces apoptosis, leading to myocyte necrosis, which facilitates the release of intracellular constituents into the bloodstream, including K^+^, PO_4_^3-^, urates, and intracellular proteins such as myoglobin, CK, LDH, AST, ALT, and other substances (Chavez et al., 2016). Their toxic accumulation may damage capillaries and contribute to fluid stasis and edema within the muscles. Damaged and ischemic tissues exacerbate metabolic acidosis and further electrolyte disturbances, amplifying the destructive process (Bagley et al., 2007; Torres et al., 2015; Zimmerman and Shen, 2013). Myoglobin, when present at concentrations that exceed the binding capacity of plasma proteins, enters the kidneys and obstructs the renal tubules, thereby promoting the development of AKI. Additionally, renal failure is further facilitated by vasoconstriction, hypovolemia, and the direct toxic effects of myoglobin on the kidneys, including effects mediated by free radicals (Chavez et al., 2016; Zutt et al., 2014b). Iron ions released during myoglobin degradation participate in the Fenton reaction, reacting with hydrogen peroxide (H_2_O_2_) to produce reactive oxygen species, which promote lipid peroxidation of cell membranes and secondarily compromise the integrity of renal tubules. The nephrotoxicity of myoglobin is further intensified by metabolic acidosis and acidic urinary pH, conditions that favor the formation of renal casts and obstruction of the distal renal tubules (Chavez et al., 2016; Zutt et al., 2014b). The main factors of the rhabdomyolysis pathomechanism are presented in Figure 2 in a synthetic manner.

### Acute Kidney Injury

The most serious consequence of rhabdomyolysis is acute kidney injury. It may occur as a result of several mechanisms associated with intense physical exertion (Chavez et al., 2016). The primary factor is hypovolemia caused by sweating, electrolyte loss—particularly under conditions of high ambient temperature, a reduced renal blood flow, and vasoconstriction of renal vessels, together with myoglobinemia/myoglobinuria (Petejova and Martinek, 2014). Typical electrolyte disturbances with metabolic consequences include metabolic acidosis, hyperkalemia (K⁺ ≥ 5.5 mmol/L), hypocalcemia (Ca^2^⁺ ≤ 2.25 mmol/L), hyperuricemia (C_5_H_4_N_4_O_3_ > 6.8 mg/dL), hyponatremia (Na⁺ < 135 mmol/L), and hyperphosphatemia (PO_4_^3-^ > 1.6 mmol/L). These abnormalities may lead to cardiac arrhythmias and cardiac arrest (Bosch et al., 2009; Petejova and Martinek, 2014). Uric acid released from damaged cells can, in the acidic environment of urine, form crystals and further contribute to obstruction of renal tubules (Shimada et al., 2011).

Acute kidney injury is defined as a clinical condition characterized by a significant deterioration in renal function, with symptoms ranging from a minimal increase in serum creatinine levels to anuric renal failure. The term AKI was recently proposed to reflect the full spectrum of acute renal failure (ARF) and is characterized as a sudden (within 48 h) decline in renal function, defined as an absolute increase in serum creatinine of >0.3 mg/100 ml or >50% above baseline values, or oliguria <0.5 ml/kg/h for >6 h (Devarajan, 2008). Post-exercise AKI is considered to be primarily transient in nature (Chapman et al., 2019; Schlader et al., 2019). This condition may persist for <3 days, in which case it is classified as transient; if it lasts for 3 or more days, it is considered persistent. It is generally reversible, although it may be fatal both during the acute phase as well as in the context of an increased risk of CKD, defined according to KDIGO (*Kidney Disease: Improving Global Outcomes*) guidelines as a state of progressive loss of renal function lasting more than 90 days (Levey et al., 2005). Repeated transient episodes of AKI are believed to contribute to nephropathy affecting renal tubules and glomeruli, leading to their remodeling and to long-term functional impairment, ultimately progressing to CKD (Chawla and Kimmel, 2012; Roncal-Jimenez et al., 2016; Schlader et al., 2019, 2019).

Epidemiological studies indicate that AKI develops in 10–40% of patients with rhabdomyolysis, and up to 15% of all AKI cases can be attributed to rhabdomyolysis. High serum myoglobin levels released from damaged muscle, approximately 3865 ng/ml, are considered a good predictor of rhabdomyolysis-induced acute renal failure (ARF) (Kasaoka et al., 2010), which often requires renal replacement therapy (hemodialysis). ARF was once defined as a sudden decline in renal excretory function that prevents the maintenance of homeostasis. Today, in accordance with KDIGO guidelines, this condition is classified as AKI. The presence and severity of AKI are diagnosed based on criteria established by international working groups (Schlader et al., 2019), including the *Acute Kidney Injury Network* (AKIN) (Mehta et al., 2007a), the *Acute Dialysis Quality Initiative* (ADQI) (Bellomo et al., 2004), KDIGO (Ostermann et al., 2020), and others. Despite some differences in identification and severity classification, acute deterioration of renal function remains the unifying characteristic across these groups. Despite its name, the current definition of AKI still relies on markers of decreased renal function rather than markers of injury. This leads to delayed diagnosis, as the condition is still identified based on serum creatinine criteria only when the severity of injury is sufficient to reduce eGFR. However, the degree of the eGFR decline associated with an increase in serum creatinine of 0.3 mg/dl may range from ≥1 to ≥36 ml/min/1.73 m^2^, depending on the baseline eGFR. This very broad range of functional impairment encompassed by the term AKI further underscores the need for injury biomarkers that would allow the diagnosis of AKI in patients with milder functional decline (Martin-Cleary et al., 2023).

## Biomarkers of Kidney Injury during Physical Exercise

Acute kidney injury is the most severe consequence of rhabdomyolysis following intense physical exertion and is likewise triggered by extremely severe tissue damage. This condition results in impaired renal function, leading to the accumulation of metabolic waste products in the blood and hindering the body’s ability to maintain proper fluid and electrolyte balance (Kellum et al., 2021). When assessing the diagnostic usefulness of biomarkers, their applicability must be considered across a broad spectrum. An ideal biomarker is expected to be sufficiently sensitive and specific, to remain elevated for a period adequate for establishing a diagnosis, to increase proportionally with the degree of kidney injury, to enable prediction of clinical outcomes, and to be associated with a well-defined biological mechanism.

### Classical (Traditional) Biomarkers

Until now, the diagnosis of kidney injury has relied on biomarkers of renal function—the so-called classical markers—specifically urine output, the GFR, and serum concentrations of creatinine and urea. However, their diagnostic response is delayed relative to the onset of injury. Creatinine is a derivative of creatine, and its concentration depends on muscle mass. In the kidneys, it undergoes glomerular filtration and, to a lesser extent, tubular secretion. Serum creatinine concentrations are an insensitive marker of acute kidney injury, and the rate at which its level rises in comparison to baseline depends on the severity of the acute injury and the pre-existing functional renal reserve (Waring and Moonie, 2011). Functional changes in the kidneys, quantitatively expressed by the GFR, are widely accepted as an indicator of renal function in both health and disease, as well as of the urine production rate (Kellum et al., 2021). However, these measures appear inadequate in cases of renal failure and injury induced by intense physical exertion. Acute kidney injury may not manifest with a detectable rise in serum creatinine for at least 24–48 h after the initial injury (Waring and Moonie, 2011).

In practice, the GFR is most commonly estimated from serum creatinine using equations that correct for age, sex, race, and body mass (Bakońska-Pacoń, 2006; Beierwaltes et al., 2013), reported as the eGFR. During intense physical activity, dehydration, and prolonged exertion—particularly when oliguria or anuria is observed—an increase in serum creatinine appears to be an expected change. Until normal kidney function is restored, creatinine cannot be reliably used for diagnostic purposes. It is considered a late marker of kidney function rather than injury (Błazucka, 2019; Zdziechowska et al., 2020). Thus, an increase in serum creatinine, which normally reflects a reduction in the GFR, does not always indicate kidney injury (Parikh and Coca, 2010). For this reason, diagnostic criteria for kidney injury that rely solely on serum creatinine concentration and the urine flow rate should be applied only after optimal rehydration of the patient (Mehta et al., 2007b).

### Novel Biomarkers

Given the complexity of the physiological changes that occur after exercise rhabdomyolysis, relying solely on functional assessments of the kidneys does not allow rapid diagnosis of renal injury. Hence, there is a need to search for additional biomarkers that meet criteria of specificity and high sensitivity, that signal kidney injury at an early stage, and that are effective in predicting the risk and severity of AKI before clear functional impairment becomes detectable (Griffin et al., 2020; Malhotra and Siew, 2017; Schrezenmeier et al., 2017; Wołyniec et al., 2020).

Traditional biomarkers of kidney injury and function are unfortunately often unreliable. Their limited diagnostic value also stems from their variability depending on age, sex, muscle mass, and hydration status (Kokkoris et al., 2013). Therefore, researchers are focused on identifying new biomarkers capable of reflecting stress responses occurring at molecular and cellular levels, that are specific, and that appear much earlier—long before creatininemia develops. The evaluation of early kidney biomarkers may provide important insights into the relationship between changes in renal function and the potential risk of acute kidney injury.

In recent years, many studies have described potential new markers—referred to as renal troponins (Oh, 2020; Ortega and Heung, 2018), which hold substantial promise for diagnostic applications, although each of them also has certain limitations. These biomarkers are released by different components of the nephron and can be measured in urine samples. Novel biomarkers of kidney injury include neutrophil gelatinase-associated lipocalin (NGAL), kidney injury molecule-1 (KIM-1), liver-type fatty-acid-binding protein (L-FABP), and interleukin-18 (IL-18) (Zdziechowska et al., 2020). Some of them can already be determined using available analytical assays (Oh, 2020).

A second group of biomarkers comprises functional markers that indicate metabolic or physiological stress. The most widely studied include cystatin C, as well as cell-cycle arrest biomarkers such as tissue inhibitor of metalloproteinase-2 (TIMP-2) and insulin-like growth factor-binding protein 7 (IGFBP-7), along with calprotectin (Heller et al., 2011; Zhou et al., 2016). Additional candidates include metabolic, proteomic, and genetic biomarkers (e.g., microRNAs) (Almulhim, 2025).

### NGAL

NGAL is the most extensively studied biomarker of AKI (Zhou et al., 2016). It is a small 25-kDa protein covalently associated with neutrophil gelatinase. Expression of this protein increases in renal injury caused by ischemia-induced hypoxia or exposure to nephrotoxins. It is synthesized in distal tubular cells, the ascending limb of the loop of Henle, collecting ducts, and the epithelial cells of the proximal tubules (Schrezenmeier et al., 2017). NGAL has the ability to bind iron and is an important factor in the generation of free radicals. An antioxidant role has also been suggested, indicating potential protection against oxidative stress-induced damage (Roudkenar et al., 2008).

Acute injury to proximal tubules leads to the rapid appearance of NGAL in urine approximately 2 h after the insult, followed by its rise in blood. It is considered the most strongly induced renal protein following ischemic or nephrotoxic injury, as shown in animal models (Mishra et al., 2003). In another study, elevated NGAL levels also appeared very early. They were detectable 3 h after injury and depending on severity, reached peak between 6 and 12 h. High concentrations in both urine and blood may precede the diagnostic rise in creatinine by 1–3 days. NGAL is the most extensively described biomarker and carries a substantial diagnostic promise. However, it is not always specific for kidney injury. It has been shown to be effective in detecting kidney injury in patients with various pathological conditions (Zhou et al., 2016). Its levels may increase during inflammatory states (Devarajan, 2010; Monari et al., 2020). Its concentration is significantly higher in AKI than in CKD, which is why it is considered a very useful early predictive biomarker of AKI, as well as a valuable biomarker of early renal recovery in AKI (de Geus et al., 2012; Devarajan, 2008).

### KIM-1

KIM-1 is a type 1 transmembrane glycoprotein with a molecular weight of 38.7 kDa and an extracellular immunoglobulin domain. Its expression in a healthy kidney and urine is undetectable. Strong KIM-1 expression is observed after ischemic or toxic injury, primarily in the proximal tubular cells of the nephron. During injury, the extracellular domain of this protein is shed from the cell surface in a metalloproteinase-dependent process and subsequently excreted in the urine (Schrezenmeier et al., 2017). Increased urinary levels of the extracellular domain are detected immediately after AKI (Devarajan, 2008). KIM-1 is believed to play an important role in epithelial repair processes and in the removal of dead cells from the tubular lumen. It may serve as a useful biomarker of proximal tubular injury and of adverse clinical outcomes (need for dialysis or death) in patients with AKI, as it has been shown to function as a phosphatidylserine receptor and thus mediate phagocytosis of apoptotic bodies and cellular debris. It has been proposed that KIM-1 is important for kidney and tubular regeneration, as demonstrated in cultured renal epithelial cells (Ichimura et al., 2008).

KIM-1 levels increase significantly within a few hours after the injurious stimulus. They may also rise during urinary tract infections, and diagnostic performance varies depending on the etiology of AKI. Urinary KIM-1 concentrations have been shown to predict adverse outcomes, the need for dialysis, and mortality (Liangos et al., 2007). It is considered a highly reliable marker of nephrotoxicity in preclinical studies. Rapid dipstick tests for urinary KIM-1 are already available, enabling broader application across different patient populations and facilitating further preclinical and clinical diagnostics. Elevated KIM-1 concentrations also provide opportunities for CKD diagnosis (van Timmeren et al., 2007).

An important advantage of this biomarker is its specificity for ischemic kidney injury and the availability of a rapid analytical assay. KIM-1 and NGAL are regarded as excellent urinary and blood biomarkers for early prediction of AKI. Similarly, the diagnostic usefulness of TIMP-2 and IGFBP-7 in urine is considered very favorable for the detection and staging of moderate to severe AKI (Wasung et al., 2015). According to these authors, the combined measurement of creatinine and cystatin C provides a more accurate estimation of the GFR.

### IL-18

Interleukin-18 is a pro-inflammatory cytokine with a molecular weight of 24 kDa, secreted by antigen-presenting cells, including macrophages. It is also known as an interferon-γ-inducing factor. It is initially synthesized as an inactive precursor lacking a signal peptide and remains inside the cell until cleaved by caspase-1, a component of the inflammasome—a protein complex involved in the maturation and release of interleukins in response to external stimuli (Schrezenmeier et al., 2017). IL-18 is a sensitive marker of ischemic kidney injury, but also of injury in other organs, including the brain and the heart. In the nephron, it is primarily expressed in the distal convoluted tubules, connecting tubules, and the collecting system. In the study of Parikh et al. (2006), IL-18 concentrations increased 3–6 h after injury, peaked around the 12^th^ h—reaching more than 25-fold elevations—and remained elevated for more than 48 h in patients undergoing cardiac surgery. Its role in kidney injury involves the infiltration of neutrophils into the renal parenchyma (Parikh et al., 2006).

### L-FABP

L-FABP is a small lipid-binding protein of 14 kDa, originating from the liver. In the kidney, it is primarily located in the proximal tubules, where it is secreted into the tubular lumen along with bound toxic peroxisomal products. Activation of the peroxisome proliferator-activated receptor α (PPAR-α) enhances the expression of the L-FABP gene (Landrier et al., 2004). This protein participates in the regulation and uptake of fatty acids as well as their intracellular transport to mitochondria and peroxisomes (Chmurzyńska, 2006), where β-oxidation provides cellular energy. Furthermore, L-FABP contributes to the protection of cells against oxidative stress induced by ROS, including H_2_O_2_ (Wang et al., 2005). Therefore, it is considered an important intracellular antioxidant during oxidative stress, maintaining low concentrations of free fatty acids in the cytoplasm of tubular cells by facilitating their intracellular metabolism and urinary excretion (Pavlakou et al., 2017). Its expression increases during renal hypoxia. Patients with high L-FABP levels at ICU admission showed a greater risk of developing AKI within one week (Doi et al., 2011). Thus, L-FABP may identify patients with high susceptibility to renal stress (Schrezenmeier et al., 2017).

### TIMP-2 and IGFBP-7

TIMP-2 and IGFBP-7 are the so-called *cell-cycle arrest biomarkers*. According to Ortega and Heung (2018), this phenomenon may represent a cellular escape mechanism that protects cells from sustaining further damage; cell-cycle arrest in the G1 phase may therefore serve as a protective response in situations where potential DNA injury could occur. Such conditions include renal ischemia or oxidative stress. Under these circumstances, renal epithelial cells undergo G1 cell-cycle arrest (Ortega and Heung, 2018). In 2013, IGFBP-7 and TIMP-2 were identified as AKI biomarkers (Kashani et al., 2013). TIMP-2 is a 21-kDa protein belonging to the family of tissue inhibitors of metalloproteinases. IGFBP-7 is a 29-kDa protein that binds to IGF-1 receptors (Evdokimova et al., 2012) and inhibits their activation by IGF-1. The cellular sources of IGFBP-7 and TIMP-2 in AKI are poorly understood. Apart from the report identifying elevated urinary levels of IGFBP7 and TIMP-2 in patients at risk of AKI, the exact sites of synthesis of these molecules during AKI remain unknown. Although Kashani et al. (2013) speculated that IGFBP-7 and TIMP-2 were synthesized by renal tubular cells, there is currently no scientific evidence confirming this hypothesis (Schrezenmeier et al., 2017).

It has been demonstrated that the diagnostic value of the combined product [TIMP-2]·[IGFBP-7] is significantly higher than that of each biomarker alone in predicting AKI. Based on two large clinical studies (Devarajan, 2008; Guzzi et al., 2019), [TIMP-2]·[IGFBP-7] has been shown to be a useful functional biomarker for predicting severe AKI within 12 h. In a healthy patient population, [TIMP-2]·[IGFBP-7] showed no significant sex-related differences but exhibited a weak inverse correlation with age (Chindarkar et al., 2016).

Among the numerous modern biomarkers used for diagnosing kidney injury, only ([TIMP-2]·[IGFBP-7]) and NGAL—measured in urine or plasma—are supported by a substantial body of clinical evidence. In diagnostic practice, at least three commercially available immunoassays are used to facilitate early AKI detection or identify short-term kidney injury risk, thereby enabling earlier diagnosis and treatment as well as improving clinical outcomes. NephroCheck® (Astute Medical, Inc., San Diego, California, USA) assesses urinary [TIMP-2]·[IGFBP-7]. Additionally, urinary NGAL can be measured (ARCHITECT®, Abbott Laboratories, Abbott Park, IL, USA), as well as urinary and plasma NGAL (BioPorto Diagnostics A/S, Hellerup, Denmark) (Brazzelli et al., 2022; Guzzi et al., 2019; Wołyniec et al., 2020).

There are also other biomarkers, including tubular enzymes, of which presence in the blood indicates specific sites of injury. These include N-acetyl-β-D-glucosaminidase (NAG), gamma-glutamyl transpeptidase (GGT), and glutathione-S-transferase (GST), which are present in proximal tubules and of which activity increases primarily after exposure to nephrotoxins (Almulhim, 2025), as well as after physical exercise (Bakońska-Pacoń and Borkowski, 2003; Luft, 2021; Wołyniec et al., 2020).

It has been shown that useful indicators of exercise-induced physiological changes detectable in urine may include lysosomal enzymes, e.g., arylosulfatase (ASA), β-glucuronidase (β-GRS) or NAG and brush-border enzymes, e.g., γ-glutamyltransferase (GGT), leucyloaminopeptidase (LAP) and alanine aminopeptidase (AAP). In post-exercise urine analysis, as previously discussed, increased concentration and temporal dynamics of total protein, albumin, low-molecular-weight proteins such as β_2_-microglobulin, RBP, lysozyme, and soluble adhesion molecules (sVCAM-1, sICAM-1, E-selectin, sP-selectin), extracellular matrix proteins such as fibronectin and laminin, as well as E-cadherin (a protein involved in cell adhesion), can be evaluated. Changes in these variables reflect the generalized systemic response to stress induced by physical exertion, while urinary alterations may also indicate changes occurring within the glomeruli and renal tubules.

Many additional substances and proteins are being investigated for potential usefulness in the diagnosis of kidney injury; however, their detailed description exceeds the scope of this study. Interested readers are referred to appropriate sources (e.g., Almulhim, 2025; Bellomo and See, 2021; Brazzelli et al., 2022; Devarajan, 2008; Luft, 2021; Mistry et al., 2025; Tidmas et al., 2022). In this work, we focused only on the most widely studied biomarkers that are already approved for commercial testing or have strong potential for future implementation.

Despite promising results, most novel biomarkers of kidney injury—although well-established in scientific research—have not yet entered routine clinical practice (Almulhim, 2025). There remains a need to determine how these biomarkers can optimally be used in patient management and whether their application improves clinical outcomes. Moreover, diagnostic thresholds for different populations must be established, analytical validation of laboratory assays needs to be completed, and the effects of non-renal factors on biomarker levels should be clarified. Their role relative to traditional markers such as serum creatinine must also be more clearly defined (Almulhim, 2025).

## Conclusions

Many authors emphasize that new early kidney biomarkers should be introduced into diagnostic practice with great caution. Early clinical diagnosis of kidney injury based on urinary or plasma biomarkers still poses numerous challenges, particularly when the etiology of AKI is not well understood. Misinterpretation of biomarker test results may lead to inappropriate clinical decisions, potentially harming patients (Vanmassenhove et al., 2013), including individuals hospitalized following excessively intense physical exertion. Therefore, even though many new kidney biomarkers exist and appear highly promising, their implementation into routine clinical practice should proceed slowly and with great care.

The absence of conclusive studies on novel biomarkers in the context of physical exercise also indicates the need for prospective research. Such studies should focus on large and heterogeneous cohorts of athletes. Furthermore, they should encompass a wide range of sports disciplines in order to determine the dynamics of changes in biomarker concentrations across different contexts, including pre- and post-exercise and post-training conditions; during competitive, pre-competitive, and recovery periods; as well as under varying environmental conditions. Systematized knowledge of renal injury biomarkers after exercise, combined with education of coaching and medical staff, should contribute to ensuring training safety, promoting rapid physiological recovery, and facilitating athletes’ timely return to sports competition.
